# Data Stream Mining Applied to Maximum Wind Forecasting in the Canary Islands

**DOI:** 10.3390/s19102388

**Published:** 2019-05-24

**Authors:** Javier J. Sánchez-Medina, Juan Antonio Guerra-Montenegro, David Sánchez-Rodríguez, Itziar G. Alonso-González, Juan L. Navarro-Mesa

**Affiliations:** 1 Centro de Innovación para la Sociedad de la Información (CICEI), Universidad de Las Palmas de Gran Canaria, Campus Universitario de Tafira, 35017 Las Palmas de Gran Canaria, Spain; juanantonio.montenegro@ulpgc.es; 2 Instituto Universitario para el Desarrollo Tecnológico y la Innovación en Comunicaciones, Universidad de Las Palmas de Gran Canaria, Campus Universitario de Tafira, 35017 Las Palmas de Gran Canaria, Spain; david.sanchez@ulpgc.es (D.S.-R.); itziar.alonso@ulpgc.es (I.G.A.-G.); juanluis.navarro@ulpgc.es (J.L.N.-M.)

**Keywords:** short-term wind speed prediction, data stream mining, extreme weather forecasting, adaptive learning, linear regression, sensor network, touristic destinations

## Abstract

The Canary Islands are a well known tourist destination with generally stable and clement weather conditions. However, occasionally extreme weather conditions occur, which although very unusual, may cause severe damage to the local economy. The ViMetRi-MAC EU funded project has among its goals, managing climate-change-associated risks. The Spanish National Meteorology Agency (AEMET) has a network of weather stations across the eight Canary Islands. Using data from those stations, we propose a novel methodology for the prediction of maximum wind speed in order to trigger an early alert for extreme weather conditions. The methodology proposed has the added value of using an innovative kind of machine learning that is based on the data stream mining paradigm. This type of machine learning system relies on two important features: models are learned incrementally and adaptively. That means the learner tunes the models gradually and endlessly as new observations are received and also modifies it when there is concept drift (statistical instability), in the modeled phenomenon. The results presented seem to prove that this data stream mining approach is a good fit for this kind of problem, clearly improving the results obtained with the accumulative non-adaptive version of the methodology.

## 1. Introduction

The Canary Islands are a world-class tourist destination for many reasons, namely their accessibility from Europe, their European-standard services, and the hospitality of the locals after decades of tourism being their main industry. According to the Canary Island Statistics Institute (ISTAC), tourism makes up nearly 35% of all the economic activity in the Canary Islands.

Another crucial element for the attractiveness of this destination is the year-round temperate climate. However, there are exceptions to this benevolent climate. There have been episodes of extreme weather where, to some degree, the safety of the population and the local economy has been compromised. Tropical storm Delta [1,2] in November 2005, for instance, cost a loss of up to 364 million USD and at least 7 direct fatalities [3]. Over 225,000 residents experienced power outages and 12,000 lost telephone services. The peak gust recorded in the island of La Palma was 95 mph (152 km/h), and in Tenerife the maximum gust was 90 mph (147 km/h).

According to [4], there is strong evidence of the link between climate change, especially anthropogenic warming, and an increase in extreme rainfalls and the wind speed of tropical cyclones. A positive trend in the number of Atlantic tropical cyclones has been observed since 1990, although it is not possible to directly extrapolate those results to the Canary Islands’ local conditions.

In addition, there is not a large corpus of research on climate change implications in the Canary Islands. In [5] for instance, there is no clear evidence of an increase in extreme rainfall for the whole archipelago as a consequence of climatic change. However, in [6], the authors found a clear, strong correlation between extreme strong wind episodes for an area including both the Canary Islands and the nearby archipelago of Cape Verde.

Therefore, it would seem desirable to improve the Canary Islands’ resilience to extreme weather; one way to do this may be through early alert predictive modeling tools. This work presents a new methodology for predicting maximum wind speed using a sensor network deployed in the Canary Islands that consists of the 68 AEMET weather stations.

In recent years, big data and data science are permeating many research areas due to the accelerated growth of data availability and the development of data warehousing architectures and data mining methodologies. One of these fields is weather forecasting, which has been an active research topic in recent years mainly because of its deep implications in the economy, safety, and the environment.

With regard to wind forecasting, many approaches have been designed based on traditional machine learning algorithms, using techniques such as probabilistic modeling [7], decision trees [8], or advanced deep learning [9], to name a few. Nevertheless, most of these weather data usually come continuously in the form of high speed and high volume data streams. There are at least two reasons why that traditional data mining is not an optimal fit to model such phenomena. When applying the traditional off-line model learning paradigm, separating a training and testing data-set (sometimes also a cross-validation data-set), huge amounts of data have to be collected for a long period of time, assuming both the statistical stability of the phenomenon to be modeled and an infinite available computing power (memory and CPU). This is not very realistic in most cases because that approach does not seem capable of efficiently analyzing an increasing amount of data [10]. Thus, there is an alternative, newer approach that we can call online machine learning or data stream mining, where the model learning is incrementally performed using a prequential learning strategy [11].

The main objective of this paper is to share research done around the use of adaptive and incremental machine learning strategies to predict the average maximum wind speed (VMAX10m) in a 60 min horizon, in a way that is reliable and robust enough to cope with the diversity of the geographical position of each station in the region. In order to do this, the predictive modeling task was tackled through a data-stream-mining-based methodology, which means the models to be developed are learned incrementally and can adapt to the stochastic instability of the process to be modeled, in this case, maximum wind speed.

The present work is framed within the ViMetRi-MAC (“Sistema de vigilancia meteorológica para el seguimiento de riesgos medioambientales”, is funded under Programa de Cooperación Territorial. INTERREG V A España-Portugal. MAC 2014-2020) project. It is classified in Priority axis 3, the goal of which is to improve the capacity to respond to possible natural risks that affect the Macaronesic archipelagos in the North Atlantic Ocean Area, including Madeira, Azores, Cape Verde, and the Canary Islands, with an emphasis on adaptation to climate change and prevention and risk management. The main objective of ViMetRi-MAC is promote the development of public–private synergies to address the risks linked to meteorological events potentially causing disasters. A system will be developed in real time that facilitates the optimal management of catastrophes in terms of population and territory.

The rest of this paper will be organized as follows. In Section 2, a brief discussion is included on the state-of-the-art regarding short-term wind speed prediction. In Section 3, a brief conceptual introduction to Data Stream Mining is included, followed by Section 4, which gives a description of the data-set used, together with a brief exploratory analysis of it. In Section 5, the proposed methodology is described in detail, and in Section 6, the preliminary results obtained so far are shown. In Section 7, the main results and contributions of this work are discussed. The conclusions and future work ideas are finally shared in Section 8.

## 2. State of the Art

Short-term wind speed prediction is a very active research field of special interest today when climate change and the need for greenhouse gas emission reduction are of utmost importance for many governments. For instance, in the European Union, the goals of the Europe 2020 strategy for smart, sustainable, and inclusive growth include a 20% cut in greenhouse gas emissions with respect to 1990 levels, 20% of all EU energy production coming from renewable sources, and a 20% improvement in energy efficiency [12].

An essential part of this is wind farming. Therefore, short-term wind speed prediction is an important challenge, in particular regarding the optimization of the automatic control of wind turbines. For instance, blade pitch control is essential for maintaining a given number of r.p.m. On a longer time scale, wind speed predictions with a horizon of hours may help in the slow procedure of turning turbines on and off [13].

For this task, there is an extensive corpus of literature with a focus on time series analysis: a single variable is monitored across time and its seasonality, trend, and auto-correlation are learned to build the best possible mathematical (analytical) model, typically trying to capture directly or indirectly the probability distribution of wind speed. Much research has been carried out in this area. To name a few, on the more classical analytical side, there are many autoregressive models such as [14] or those using hidden Markov models (HMMs), as in [15]. In [14], autoregressive moving average process (ARMA) and persistence models have been developed for forecasting wind speed in a 10-h horizon at five different locations by transforming and standardizing the training time series. In [15], first- and second-order discrete HMMs have been developed for forecasting wind power and have been applied to real wind power data.

In the more recent machine learning realm, there is a large segment of literature where artificial neural networks are the central methodology, such as [16,17], or [18]. In [16], Salcedo-Sanz et al. presents a variation of the hybridization of the fifth-generation mesoscale model (MM5) using neural networks for the prediction of short-term wind speed, applied to the hourly averaged wind speed at a wind park. Their ultimate goal is to predict the total power production of the wind farm.

In [17], the authors share a comparative study on the use of adaptive linear element (ADALINE), back propagation, and radial basis function for their application in 1-h-ahead wind speed forecasting. Again, hourly mean wind speed was the prediction variable, using data from two sites in North Dakota.

Feature engineering and deep learning is used for predicting ultra-short-term wind speed in [18]. Their results seem to indicate that carefully selected deep neural networks outperform classical neural networks.

Moreover, in recent works, advanced deep learning techniques based on recurrent neural networks [9] and convolutional neural networks [19] are proposed for wind power forecasting and for the forecasting of the concentration of suspended particles with a diameter equal to or less than 2.5 μm, respectively. In both cases, the experimental results showed that compared with the traditional machine learning methods, the proposed systems obtained the best forecasting.

There are also support vector machine (SVM)-based works like [20], where they compare this technique to the results obtained with a multilayer perceptron for mean daily wind speed prediction in Madina, Saudi Arabia. Experiments seem to support the superiority of SVMs in this case.

Kalman filtering is also frequently used in several works such as [13] or [21]. Kalman filtering was used in [13] to predict one-minute-average wind speeds for Stornoway in order to reduce the error of the “persistence” forecast. In [21], the authors used Kalman filtering applied to the direct output of other numerical models, correcting systematic errors.

Other, less common probabilistic techniques may be Bayesian networks like those used in [7,22], decision trees as in [8,23], or hybrid methodologies like the one in [24], where the automatic feature selection procedure is the central element.

In [7], an advanced technology of modeling dependence structures based on the regular vine copula was introduced to the field of probabilistic wind power forecasting. The model obtained good performances in both complete and missing data cases, with the added value of describing forecast conditions. In addition, in [22], a Bayesian time series forecasting model is proposed. One very interesting element in Bayesian analysis that this work benefits from is the possibility of incorporating domain expert knowledge into the models. The authors’ methodology incorporates high frequency wind speeds collected from wind turbines and takes advantage of the concept of structural breaks.

In [8], an ensemble model based on random forests was used to predict wind energy. Authors used the spatial average of the wind speed and the wind direction in addition to past power values as inputs to the system. Results showed better forecasts than classical methods such as neural networks. In addition, in [23], a hybrid between transfer learning and gradient boosting decision trees (GBDT) is proposed for developing a wind power quantile regression model. Instance-based transfer learning is a very appropriate method for the generalization of the models to different geographical sites.

A quite different approach is proposed in [24], where the researchers combine a Coral Reef Optimization (CRO) algorithm with operators from the Harmony Search in order to select the best possible meteorological attribute to train an Extreme learning machine (ELM) network.

In [25], the limitations of weather prediction models were studied. In particular, they focused on the “lateral boundary conditions” of the Canary Islands in accounting for infrequent extratropical storms such as Delta.

Without denying the merits of the works listed above, some of them quite sophisticated and accurate, no works were found that focused on developing the wind speed models incrementally and adaptively, as we propose in the present work. This seems to be an uncovered research niche since, as explained in [26], wind speed is a very random process, both in time and space, and even with the best state-of-the-art short-term wind speed models, the parameters fit for a particular location may not work well at other locations with different probability distributions. In other words, these models are not easily generalizable once trained.

**Figure 1:**
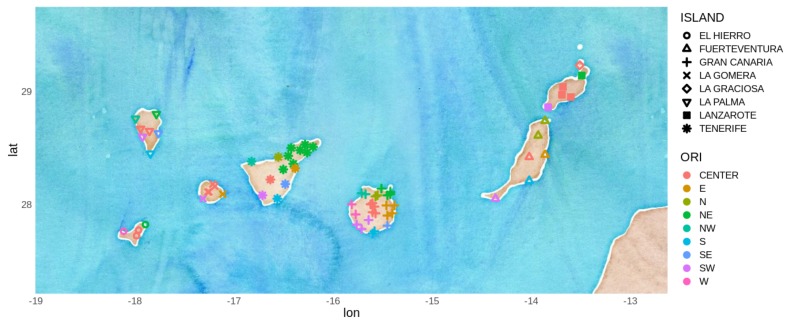
Agencia Estatal de Meteorología (AEMET) weather stations in the Canary Islands.

It seems a logical next step to develop learning models that are robust and flexible enough to be able to adapt to changing probability distributions. With the proposed methodology and the results obtained using quite geographically diverse weather stations (Figure 1 and Table 1), we aim to show that a different approach is possible through data stream mining, but at the cost of reviewing and tweaking the existing methods to work in such online setups.

## 3. Data Stream Mining

We are undergoing social and economic change driven by data. The availability of data in every aspect of our lives has become a gargantuan source of possibilities, and great efforts are put into how to automatically extract knowledge from them. The exponential acceleration of data production is driven mainly by the pervasiveness of computing (i.e., personal computing devices), the deployment of sensor networks, and global hyper-connectivity. It is the so-called Internet of Everything (IoE, [27]), and it yields big data in the 5 V’s sense: volume, velocity, variety, veracity, and value, as enunciated in [28,29].

When it comes down to volume and velocity in particular, it is clear there is a big bottleneck ahead. It is becoming increasingly impractical to store all the produced data in order to extract knowledge from it at a later date. New online methodologies need to be developed. A whole new realm of machine learning literature needs to be developed in order to build models in real time capable of extracting knowledge as new data observations are arriving in an incremental and adaptive way.

Data stream mining [30,31], this new dynamic approach to data mining, is the natural evolution of machine learning and data mining under the pressure of big data scales and more importantly, the obsolescence of the concepts modeled during the data acquisition process. This new approach has many advantages like contention in the required computational resources, or reduced lag in the learning and execution of the predictive models.

In data stream mining, a central concept explaining its very need is concept drift [32,33]. Concept drift is defined as a change in the probability distribution of the modeled variable in the data stream received. More formally, concept drift between time point *t* and time point t+1 happens when the inequality in Equation (1) is true.

(1) $$\exists t:p_{t}\left(X,y\right)\neq p_{t+1}\left(X,y\right)$$

In Equation (1), $p_{0}$ and $p_{1}$ denote the joint distribution at times *t* and t+1, respectively, between the set of input variables *X* and the target variable *y*.

In other words, there is concept drift when there is stochastic non-stationarity in the phenomenon that is being modeled. Hence, concept drift can be detected either by direct statistical methods (mean, variance, and auto-covariance) or by indirect practical methods, like observing statistically significant worsening in the performance of a previously trained model.

## 4. Data-Set Used and Exploratory Analysis

The data-set used to apply the proposed methodology has been obtained through the ViMetRi-MAC project, provided by the Spanish public agency AEMET (Agencia Estatal de Meteorología). It is a proprietary data-set, and it is not possible to redistribute it. It includes the variables measured by 68 weather stations spread across the Canary Islands as shown in Figure 1. Each station is equipped with one of the three following equivalent platforms, with firmware customized by AEMET:

- Datalogger DLx-MET
- Vaisala HydroMet System MAWS301
- SEAC EMA55

The observations used for this work were selected in a period of time from 12:10:00 on 26 April 2018 to 11:50:00 on 13 December 2018 local time, at a 10-min sampling rate. In total, 61,057,225 observations were used for the present study.

The data used are from a proprietary data set belonging to AEMET, facilitated within the ViMetRi-MAC project. The data were downloaded from their servers in files per day studied. Each weather station was equipped to compile several variables, such as max wind speed, average wind speed, temperature, humidity, precipitation, atmospheric pressure, etc.

Table 1 shows the position, latitude and longitude coordinates, and ID code (IDEMA) of each weather station. The Canary Islands (Spain) are a group of eight small islands (Gran Canaria, Tenerife, La Palma, La Gomera, El Hierro, Fuerteventura, Lanzarote, and La Graciosa) situated opposite southern Morocco, in the bounding box 29°29′08.4″*N*, 13°22′18.8″*W* and 27°43′21.7″*N*, 18°11′34.8″*W*. The population of the islands is 2,127,685, 42.5% of which is located in Gran Canaria and 39.8% in Tenerife, according to ISTAC. The main economic activity of the islands is tourism. In general, there are 24,368 companies established in Gran Canaria, 27,881 in Tenerife, and 12,135 in the other islands.

**Figure 2:**
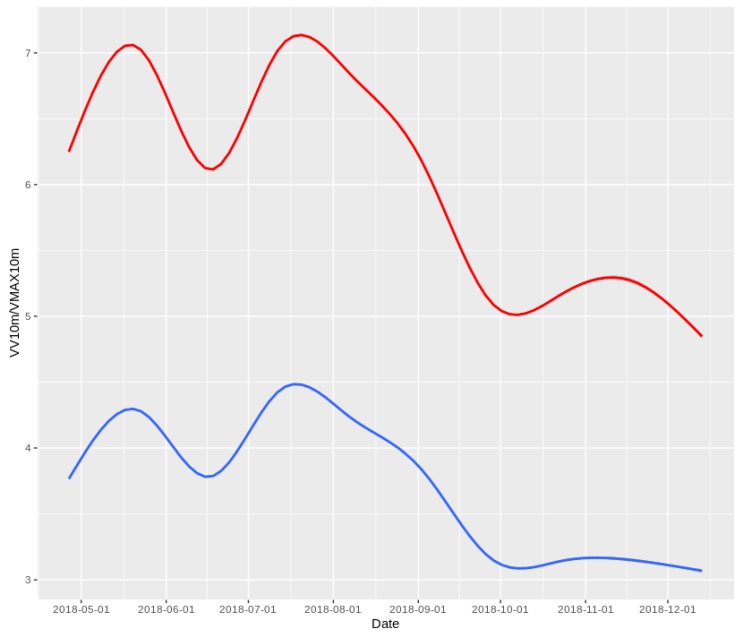
Smoothed (using GAM) evolution of VMAX10m (Maximum wind speed in red, upper part) and VV10m (averaged wind speed in blue, lower part) during the sampled period.

The variable used for wind forecasting was VMAX10m, which is the maximum speed of wind (m/s). In Figure 2 is represented both VMAX10m and VV10m, which is the average wind speed, both smoothed across the sampled period using a generalized additive model (GAM, [34]) provided by the ggplot2 library [35].

In the preliminary studies carried out with this data-set, by far the most important explanatory variable for the current maximum wind speed was the previous maximum wind speed. In other words, VMAX10m presented a very high auto-correlation level, which motivated using just that variable and previous instances of the same variable as independent variables of the proposed model. However, adding other variables to the model cannot be ruled out in order to improve its efficiency in a future expansion of this research.

**Figure 3:**
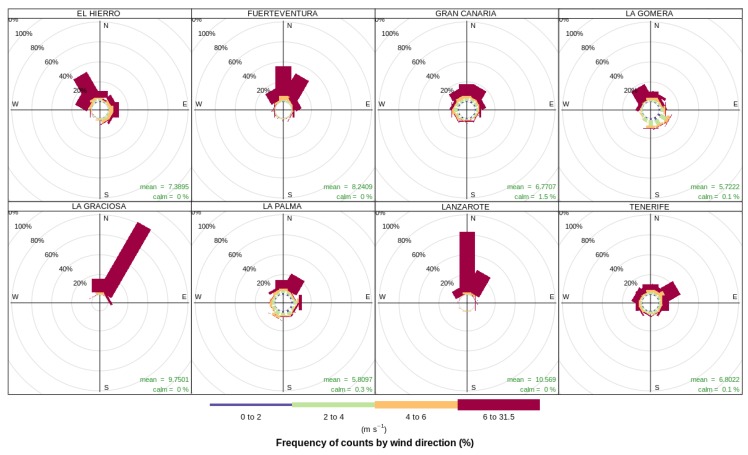
VMAX10m wind roses for each island in July.

In Figure 3 can be seen that there is a clear northern component for winds in the Canary Islands, known by the scientific community as “trade wind” for its historical implications in the Spanish and Portuguese trade with Central and South America during and after the 16th century. That figure considers only the windiest month in 2018, which was July, for each island. This image is shared to show that even when there is a predominance of northern winds in July, the wind roses for the eight islands are quite different. According to [36,37], this archipelago presents numerous micro-climates. Therefore, a model trained to predict wind has to be generalizable.

## 5. Methodology

In this research, a data stream mining methodology has been developed to predict maximum wind speed (VMAX10m) in 68 weather stations in the Canary Islands, with a time horizon of 60 min. A classic regression method, the linear regression with gradient descent, has been used as a parameter learning technique, but with modifications so that it can operate in an adaptive and incremental way.

The adaptive learning proposed below is based on the “prequential” paradigm [11]. Instead of using the classical machine (off-line) learning approach of two independent testing and training sets, in a prequential setup, the model is firstly evaluated as new observations arrive, and then re-trained with those new observations.

At this first stage of research, we have picked linear regression as a base model for the maximum wind speed prediction for two reasons. Firstly, the combination of linear regression with a learner based on gradient descent is very well known, as is its robustness. Its behavior can be easily understood and compared to other methodologies. Therefore, the modifications applied to this methodology to make it incremental and adaptable may be understood by any average reader with basic training in machine learning. Due to the lack of literature on the use of data stream mining for this application, a baseline study is needed using a very classic methodology such as linear regression and gradient descent.

The second reason is more technical. The absence of recurrence of these algorithms make them suitable for observation-based learning, and therefore easily adaptable to online training.

However, it is evident that wind speed prediction is not a linear phenomenon, and we confirm that hypothesis in a coefficient of determination analysis we have developed as part of the experiments associated to this research. The adaptability of the proposed methodology copes to some degree with the lack of linearity of the phenomenon. But, in a future stage of this research, other nonlinear base models will be adapted and tested.

The model is learned gradually as new batches of data come, so the linear regression equation parameters are modified over the modifications of previous batches of data. The forgetting strategy is implemented in two different elements. Firstly, the window size of previous instances of the VMAX10m (the dimension of the linear regression coefficient vector) is controlled. Secondly, the learning rate of the Gradient Descent algorithm is adaptively altered. Let us first consider linear regression as a modeling framework for the prediction of wind as a linear combination of previous instances of the variable VMAX10m in each weather station.

### 5.1. Linear Regression

Let it be assumed that there is a data-set ${\left\{y_{i},x_{i1},\ldots ,x_{ip}\right\}}_{i=1}^{n}$, where $y_{i}$ means the *i*-th observation of the dependent variable (to be predicted) and the respective *i*-th occurrence of all the *p* independent variables $X_{i}$.

Let it be assumed there is also a vector Θ containing p+1 values.

Then, a linear regression model can be expressed as follows:

(2) $$y_{i}=\theta_{0}1+\theta_{1}x_{i1}+\theta_{2}x_{i2}+\ldots +\theta_{p}x_{ip}+\epsilon_{i},\;i=1,\ldots ,n,$$

ϵ being the noise, disturbance, or error term, or in other words, everything that cannot be explained by the linear regression model itself.

In a matrix formulation: Y=XΘ+ϵ.

The learning process for a linear regression model consists of the minimization of ϵ. The conventional choice for the cost function is the mean squared error, which can be formulated as follows:

(3) $$\begin{array}{c} H_{\Theta }=X^{T}\Theta ,\;X=1,x_{1},x_{2},\ldots ,x_{m} \end{array}$$

(4) $$\begin{array}{c} J\left(\Theta \right)=\frac{1}{2m}\sum {\left(H_{\Theta }-Y\right)}^{2}=\frac{1}{2m}\sum_{i=1}^{m}{\left(\epsilon_{i}\right)}^{2}, \end{array}$$

*m* being the number of training examples.

Gradient Descent or Steepest Descent is a classical method for the minimization of a cost function. To be precise, it is a first-order iterative optimization algorithm, commonly used for fitting the Θ parameters in a linear regression function by minimizing *J*. Essentially, it calculates the steepest direction, or gradient, of the cost function and adjusts the model proportionally to it. In this work, Gradient Descent is used to update θ parameters as is indicated in Equation (5).

(5) $$\theta_{j}:=\theta_{j}-\alpha \frac{\delta }{\delta \theta_{j}}J\left(\Theta \right)$$

In other words, each $\theta_{i}$ parameter is updated in inverse proportion to the partial derivative of the *J* (cost) function with respect to each $\theta_{i}$. In this equation, α is the learning rate that calibrates the dimension of the step made in the direction of the gradient in the cost function *J* surface. Simply put, α weights how steepness of the cost function *J* for each dimension *i* of Θ translates into a change in the respective $\theta_{i}$.

This α parameter is typically a fixed value. In the proposed methodology, this α parameter is incremented or decremented depending on the evolution of the cost function in every execution of the gradient descent routine.

### 5.2. Adaptive Learning Strategy—Data-Stream-Mining-Based

The first part of our proposed adaptive learning methodology consists of having a variable number of θ parameters or previous instances considered in the linear regression model. When concept drift is indirectly detected by the degradation observed in the performance of the current model, the past observations window considered for the learning of the linear regression model is gradually reduced. The oldest element in that window is simply cut off, disabling that last parameter of the Θ vector when the cost (*J*) is incremented in a statistically significant amount (greater than 5%). On the other hand, when stability is detected by the improvement of the performance of the model as new observations come, one by one, more parameters or past instances of VMAX10m are added again (with the respective $\theta_{i}$ parameter initialized to 0).

The independent or explanatory variables considered in the linear regression trained model are simply the past values of the response or dependent variable to be predicted (maximum wind, VMAX10m) up to a maximum window of NMax previous values.

This means that in Equation (2), the explanatory variables matrix X is defined as follows for each station:

(6) $$X=\{1,VMAX10m_{t-H},VMAX10m_{t-(H+p)},VMAX10m_{t-(H+2p)},\ldots ,VMAX10m_{t-(H+NMax\times p)}\},$$

where *H* is the prediction horizon, *p* is the sampling time period, and NMax is the maximum number of previous epochs considered.

The second part of the proposed adaptive learning strategy consists of reducing the α learning rate parameter in Equation (5) when there is an increment of the cost function *J*. In every run of the gradient descent routine, the α parameter is reduced by a very small factor. Likewise, when the cost *J* is reduced, α is gradually incremented.

**Figure 4:**
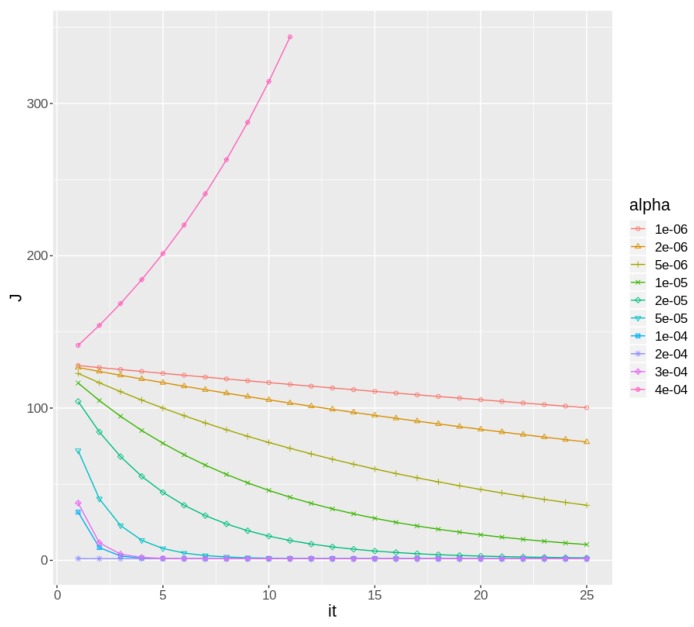
Learning curve of Gradient Descent applied to station ID C619Y during the first batch, varying α.

Figure 4 shows the learning curve corresponding to the first batch of data for weather station ID “C619Y”. It can clearly be seen how different values of α mean different behaviors of the gradient descent algorithms. Small values of α mean a slow convergence, even too slow to converge in a timely manner. And larger values may mean too large jumps in the *J* space, even preventing convergence in some cases.

Algorithm 1 describes our adaptive incremental linear regression proposal based on Gradient Descent.

**Algorithm 1:** Adaptive incremental linear regression gradient descent learner

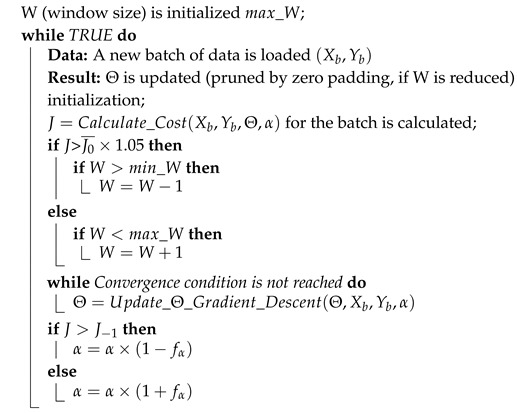



To reduce the size of the window *W*, firstly $\bar{J_{0}}$ is calculated simply by averaging a number ($W_{J}$) of previous *J* values. To be a bit more conservative, the current *J* is only considered significantly larger than the averaged $J_{0}$ if it is 5% larger.

Regarding the learning rate α, the proposed method is to reduce it by a small factor ($1-f_{\alpha }$) if the final cost *J* at the end of the Gradient Descent algorithm is larger than the *J* obtained in the previous batch, and likewise, to increment it by a small proportion ($1+f_{\alpha }$) if the cost has been reduced or stays the same, $f_{\alpha }$ being a small number between 0 and 1. That way, when there is a small deterioration of the performance of the model, the Gradient Descent algorithm will take slightly smaller steps, and when it seems like the model is improving batch after batch, α is allowed to grow slightly, to go further into convergence during the gradient descent run.

The convergence condition of the gradient descent routine is when the end of the batch is reached or the cost cannot be smaller, then the averaged previous observation-wise cost or the maximum number of iterations is reached.

### 5.3. Accumulative Strategy

As a way of comparing our results with the strategy based on data stream an alternative fixed approach has been designed. The forgetting and adaptation mechanisms are suppressed by maintaining fixed the window of past instances of the VMAX10m variable and the learning rate α value, ($f_{\alpha }=0$).

In other words, this strategy does not consider concept drift, but instead it learns the regression model incrementally by tuning the Θ parameters with all the new observations that arrive continuously.

## 6. Experimental Design and Results

As explained above, for the sake of the application of this methodology, the used observations were sampled from 12:10:00 on 26 April 2018 to 11:50:00 on 13 December 2018, Canary Island local time, at a 10-min sampling rate, gathering 61,057,225 observations in total.

The initial *W* size was set at 20, meaning 20 previous values of VMAX10m, ranging from 60, 70, 80 up to 250 min before, sampled every 10 min. The minimum window size was 3 and the maximum was 20 time lags. This means from 30 min to up to 4 h and 10 min of past VMAX10m samples would be used to predict 60 min into the future. When concept drift is indirectly detected, the number of previous instances is reduced. When there is stability, *W* is expanded to a maximum of 20.

The initial α (learning rate) value was set to $1e^{-4}$. The batch size was 100 observations. The maximum number of iterations of the gradient descent algorithm (if convergence was not achieved earlier) was set to 1000. $\bar{J_{0}}$ is calculated by averaging 10 previous *J* values ($W_{J}=10$). Finally, the $f_{\alpha }$ value is 0.01 (1 percent) for increasing or reducing the value of α during the gradient descent run.

**Figure 5:**
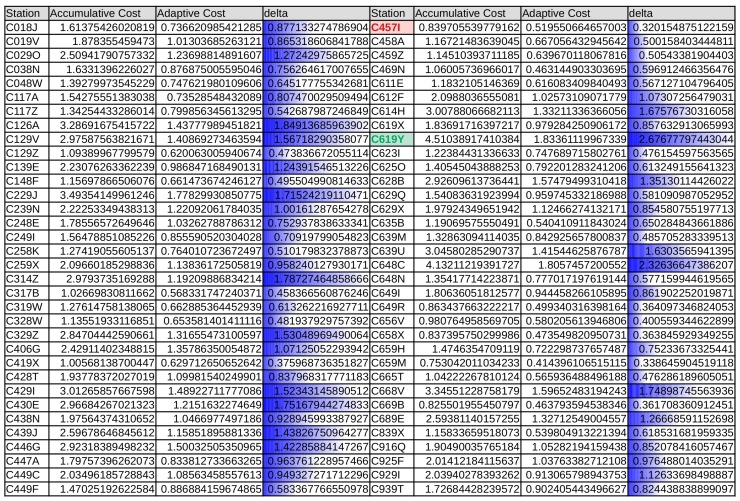
Mean accumulative and adaptive cost ((m/s)${}^{2}$) for weather stations (estimated values vs. observed values for the two strategies) and difference between the two.

The results of the application of the methodology based on data stream (“adaptive”) versus the linear “accumulative” method are presented. Both strategies were explained in the previous section. In the table depicted in Figure 5, the average cost (mean squared error, MSE) values obtained with both methodologies across the entire data-set are listed. The adaptive strategy was always superior to the accumulative strategy, as can be observed in the “delta” column (Accumulative Averaged Cost—Adaptive Averaged Cost).

**Figure 6:**
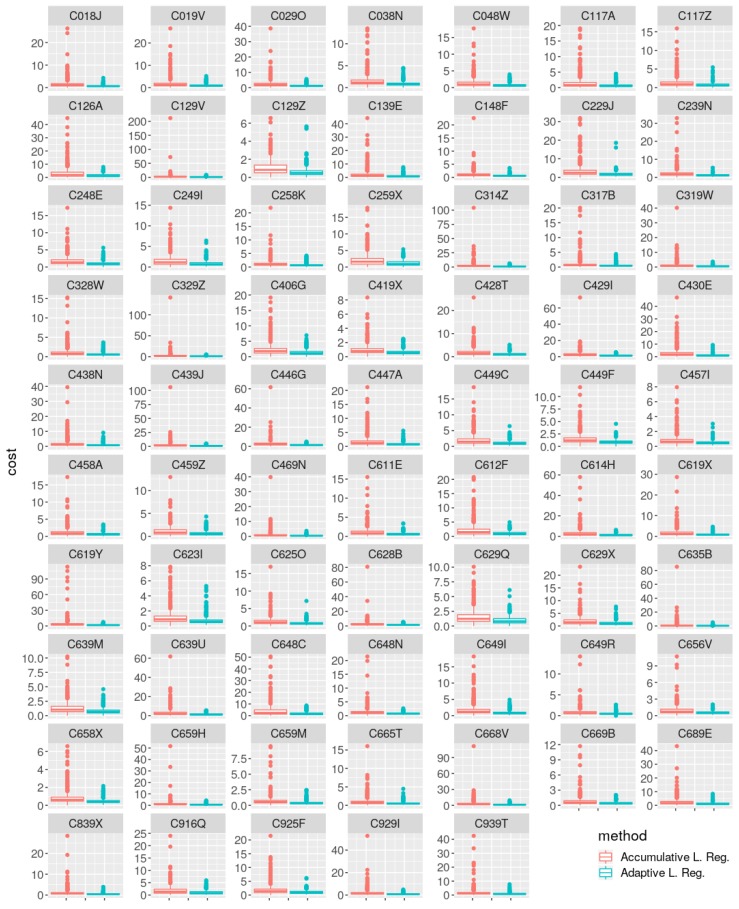
Cost (mean squared error (MSE), (m/s)${}^{2}$) box-plot comparison between both methods for each weather station.

In Figure 6, the same evidence is observed in a more visual way. Further very important evidence to point out in this analysis is about extreme cost values obtained for the accumulative strategy in comparison to the adaptive strategy. This seems to confirm the hypothesis that the adaptive methodology is able to cope much faster with concept changes than the accumulative methodology, which carries a much bigger inertia of the previous concept in its model.

**Figure 7:**
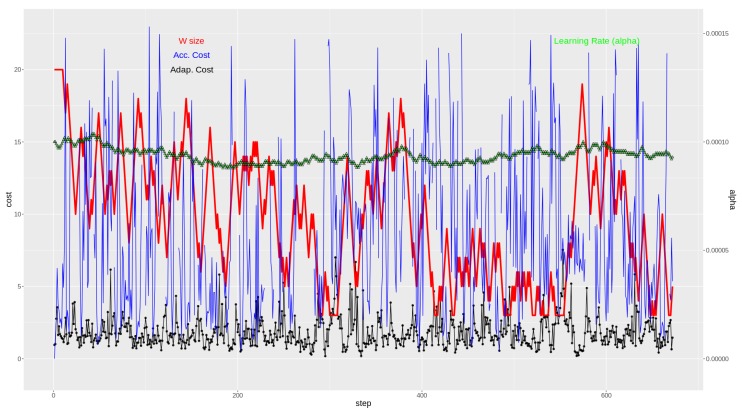
Weather station ID C619Y: Best performance case for the adaptive method. In the lower part, cost (MSE, (m/s)${}^{2}$) obtained with the adaptive strategy (black) and with the accumulative strategy (blue). In the upper part, window size (red) and learning rate (green), both for the adaptive strategy.

**Figure 8:**
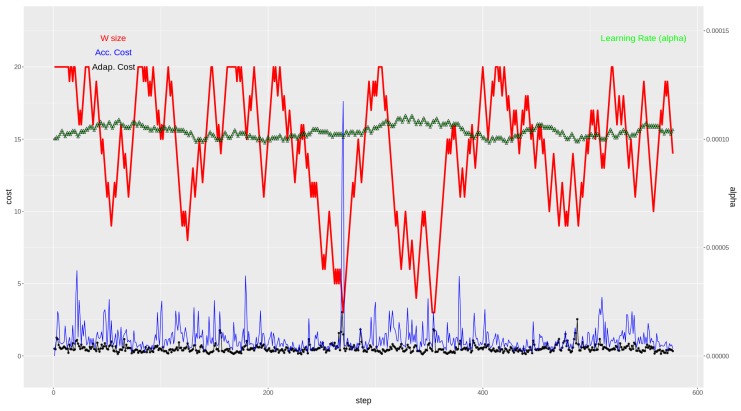
Weather station ID C457I: Worst performance case for the adaptive method. In the lower part, cost (MSE, (m/s)${}^{2}$) obtained with the adaptive strategy (black) and with the accumulative strategy (blue). In the upper part, window size (red) and learning rate (green), both for the adaptive strategy.

For a more detailed understanding of the proposed methodology, the best and worst cases’ results are shown in Figure 7 and Figure 8, respectively. In the lower part of the graphs, the evolution of the cost (MSE) value across time can seen for both methodologies. The purple (upper) curve of both Figure 7 and Figure 8 is the resulting cost value for the accumulative strategy, and in the black dotted (lower) curve, the cost value evolution for the adaptive strategy is seen.

In the upper part of the two figures (Figure 7 and Figure 8), there are two curves relating to the adaptive strategy methodology. Here, the evolution of the window *W* of previous instances of VMAX10m size is represented (in red, thicker), going from 3 previous instances to a maximum of 20 previous instances. Finally, the top part shows how the α value evolves across time (green line made up of triangles).

Note that for the graph in Figure 7, the extreme values of the accumulative strategy cost representation have been pruned for a clearer graph. See the box-plot of the weather station “C619Y” in Figure 6 for the actual range of the extreme cost calculations for that station.

In both extreme cases, it is clear how the adaptive strategy contains the mean squared error of the regression function.

**Figure 9:**
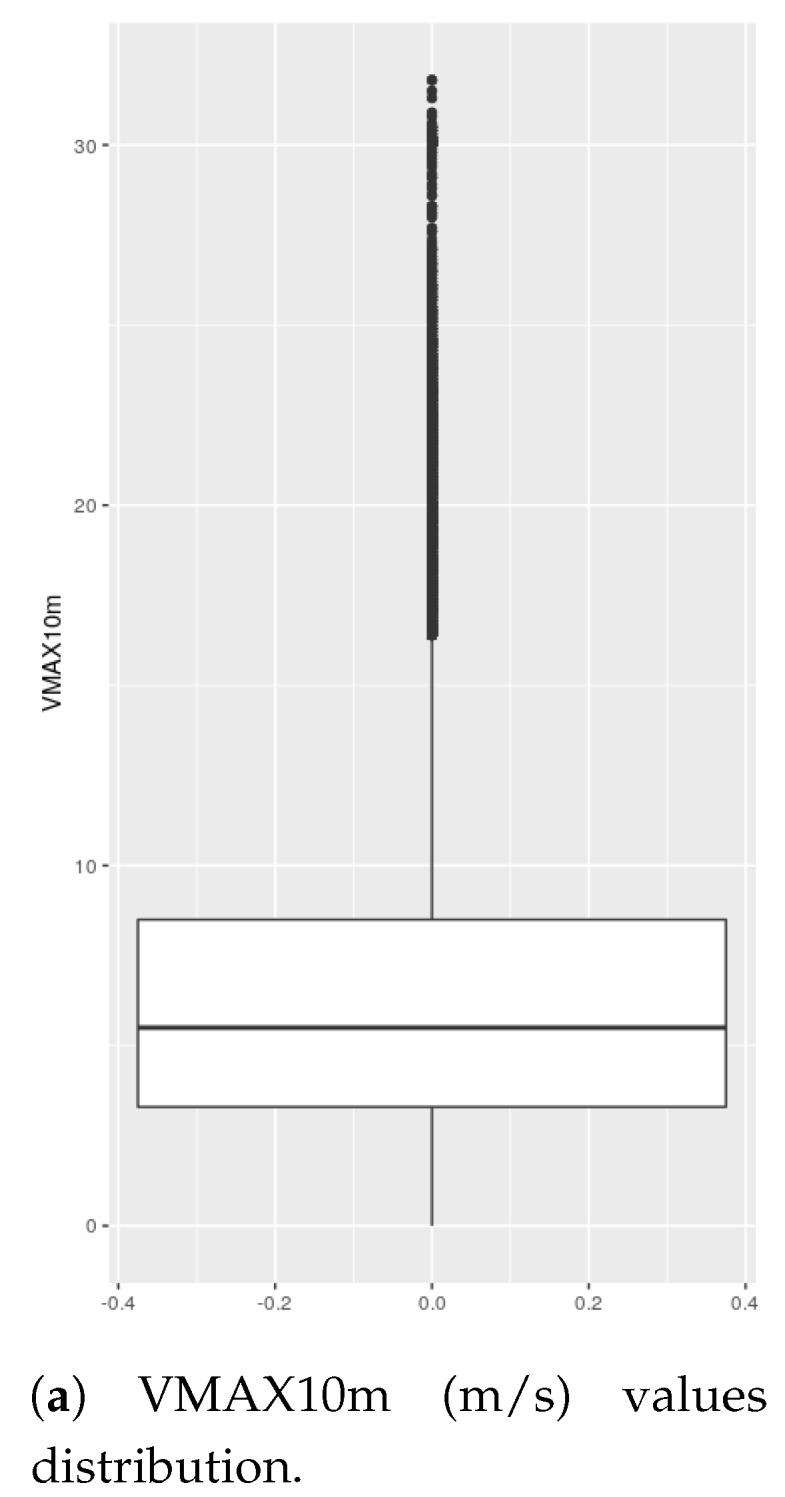

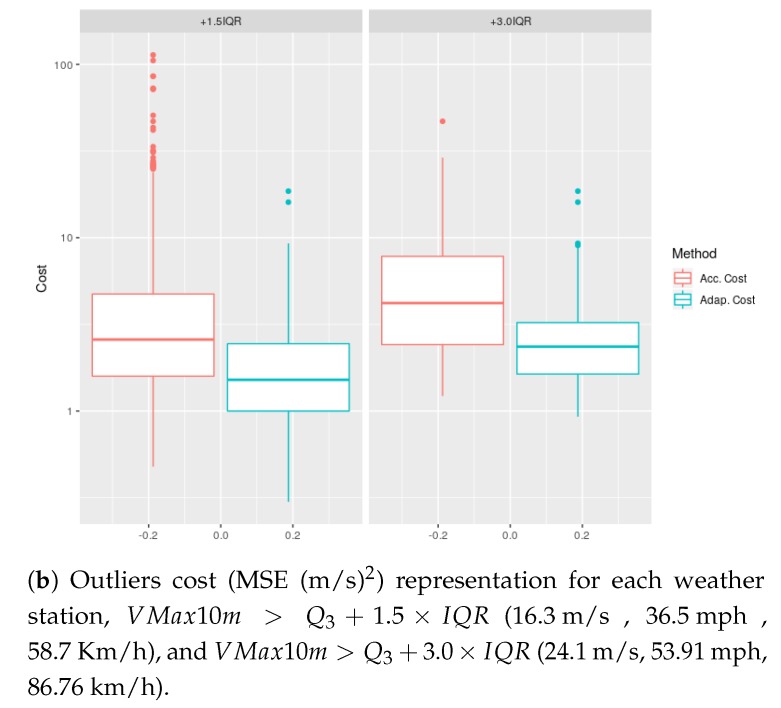
Outliers analysis (VMAX10m>16.3 m/s).

In Figure 9a, the distribution of values of VMAX10m for the entire studied period is represented. In that figure, the stronger wind speed situations have been analyzed separately, more specifically the episodes of VMAX10m above the third quartile plus 1.5 times the interquantile range and those above the third quartile plus 3 times the interquantile range ($Q_{3}+1.5\times IQR$ and $Q_{3}+3\times IQR$). For the data studied $Q_{3}=8.5$ m/s and IQR=5.2 m/s. Therefore, the two thresholds set for this analysis were VMAX10m above 16.3 m/s (36.5 mph, 58.7 Km/h) and VMAX10m above 24.1 m/s (53.91 mph, 86.76 km/h).

**Figure 10:**
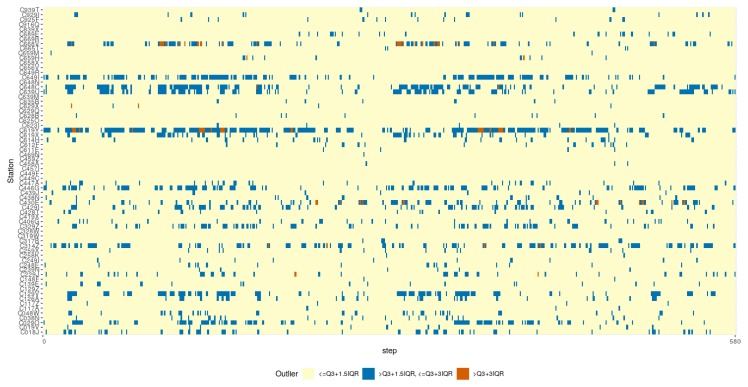
Outliers cost (MSE (m/s)${}^{2}$) representation for each weather station, $VMax10m>Q_{3}+1.5\times IQR$ (16.3 m/s, 36.5 mph, 58.7 Km/h), and $VMax10m>Q_{3}+3.0\times IQR$ (24.1 m/s, 53.91 mph, 86.76 Km/h).

In Figure 9b, the box-plot representation of the cost obtained across all of the weather stations during the outlier VMAX10m speed episodes is shown. In Figure 10, the considered VMAX10m outlier events are represented for the whole period studied. The episodes of maximum wind speed above the first threshold represent 6.39% of all observations, while the episodes of maximum wind speed above 24.1 m/s account for 0.21%.

**Figure 11:**
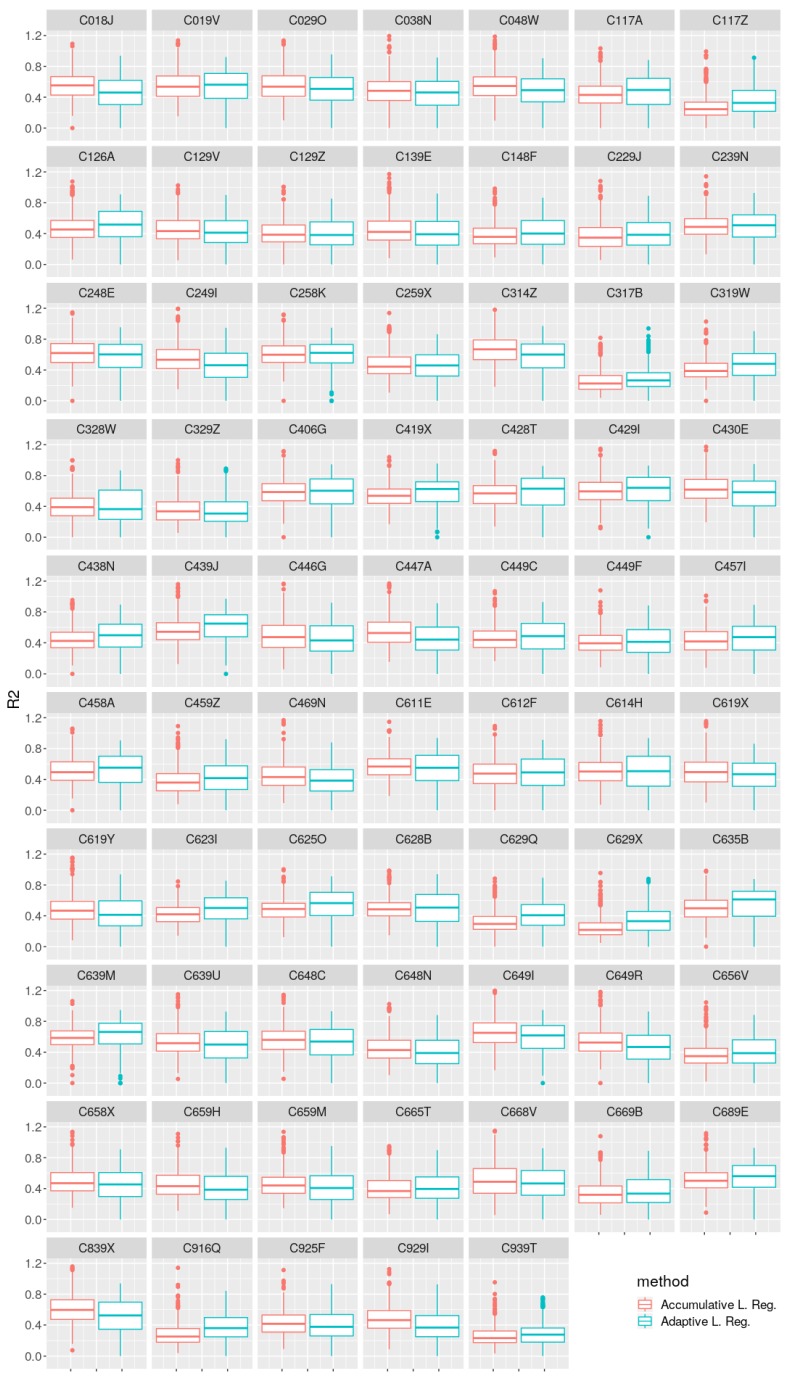
Coefficient of determination ($r^{2}$) box-plot comparison between both methods for each weather station.

Figure 11 shows the boxplot representation of the coefficient of determination Equation (7) calculated through the study period for both models. $r^{2}$ is a statistic measure that gives information about the goodness of fit of a model. It tells how much of the variance of the response variable (VMAX10m in our case) can be explained by the model, or how well the regression model approximates new observations.

(7) $$r^{2}=\frac{\sum {\left({\hat{y}}_{i}-\bar{y}\right)}^{2}}{\sum {\left(y_{i}-\bar{y}\right)}^{2}}$$

## 7. Results Discussion

In Section 6, the results of the application of the two proposed strategies, namely accumulative and adaptive, were shown. By the inspection of the average cost, calculated as the MSE of the estimated VMAX10m versus the actual VMAX10m across the entire period of study in Figure 5, it can be seen that for every case (every weather station), the obtained cost is always larger (worse) when using the accumulative strategy. This means that, even acknowledging that the results are more significant for some stations than for others, in every case, the adaptive regression model outperforms the accumulative strategy.

Figure 6 shows the cost distribution across the entire period of study. This figure is added mainly to show the distribution of outliers or extreme values of cost. Here, apart from showing the better performance of the adaptive strategy for every station, it is clear that there are much larger peaks or cost for the accumulative strategy than for the adaptive one, again for every station. This seems to be related to concept change episodes, where the previously learned concept was weighted too much in the model, negatively affecting the current concept prediction. The adaptive strategy seems more reactive to those concept changes.

If Figure 7 and Figure 8 are observed with attention, the concept changes can be indirectly detected by noting the rapid reduction of the window size (in red). In those episodes, it becomes apparent that the model built with the accumulative strategy (in blue) has a large peak in the calculated cost. However, the adaptive strategy has a much more contained behavior, seemingly due to its adaptation to the new concept to be modeled.

In Figure 9a,b, and Figure 10, the focus is placed on how the two compared strategies behave in the upper part of the VMAX10m range. Specifically, the events of VMAX10m above two defined threshold levels ($Q_{3}+1.5\times IQR$ and $Q_{3}+1.5\times IQR$) were selected, where there were maximum wind speeds above 16.3 m/s and 24.1 m/s. Everything seems to support the hypothesis that in the (infrequent) extreme wind speed conditions that this research is focusing on, again the adaptive strategy outperforms the accumulative one (Figure 9b). Looking at Figure 10, it would be possible to carry out a more detailed analysis establishing a different threshold for each station since there are obviously more frequent strong winds at different weather stations. However, the authors understand that the results obtained with this global threshold sufficiently support the conclusion that the adaptive strategy also outperforms the accumulative strategy in high wind speed regimes.

Finally, from Figure 11, a very important conclusion can be inferred that may serve as motivation for future research. A coefficient of determination above 0.6 is conventionally taken as a threshold to consider that a model explains a sufficient amount of the variance of independent variables. In the experiments carried out, however, none of the accumulative or adaptive strategies can be considered as sufficiently fit for all weather stations. Moreover, none of the strategies outperform the other in that regard. The conclusion is that, even when there is evidence showing a clear improvement in performance using an adaptive strategy, the bias of a linear regression model prevents the obtained models from explaining a sufficient portion of the variance observed. This provides a reason to extend the present research to consider adapting other nonlinear base models to work adaptively, as explained in Section 8.2.

## 8. Concluding Remarks and Future Work

### 8.1. Conclusions

In this paper, a new methodology for the prediction of maximum wind speed is proposed. This methodology uses the information of 68 weather stations spread across the Canary Islands. Our proposed methodology is special in the sense that it relies on a new style of machine learning called data stream mining, in which models are built incrementally and can be adapted to changes in the stochastic statistical distribution of the variable to be modeled.

The proposed adaptive methodology results are compared to the results obtained using a classical setup (without adaptation) of linear regression and gradient descent where only the accumulation of knowledge is considered. In other words, every new batch of observations triggers another round of gradient descent learning, re-tuning the Θ parameters continuously. This incremental approach is interesting but lacks a very important element from the data stream mining point of view because it does not have any forgetting strategy if concept drift occurs.

The results shown in Section 6 and discussed in Section 7 seem to confirm how our proposed adaptive learning strategy copes much better with the variation of the concept than the accumulative strategy. Moreover, the model learned seems to be very generalizable, since it seems to work well for the geographically dispersed network of weather stations. According to [26], transfer learning is critical for this kind of modeling task, so wind speed models can be applied to different scenarios once trained.

### 8.2. Future Research

As for future work, the plan is to compare this strategy with some other classical time series forecasting methodologies (for example, from the family of ARIMA models, support vector machines, or Kalman-filtering-based methods) that will need to be tweaked as in the present analysis with linear regression and gradient descent, in order to operate in an online setup for a fair comparison. In addition, within the realm of deep learning, other state-of-the-art machine learning techniques like recurrent neural networks should be a good comparison point with our proposed methodology. But in that case, the algorithmic modifications required to make them operative in an online setup in an incremental learning fashion will prove even harder due to the convolutional nature of most of them (loopings). It would also be possible to expand the explanatory variables used, accounting, for instance, for the altitude, the cloud cover, or how frequent strong winds are at each particular weather station, and then fusing in other directly or indirectly weather-related signals coming from other kinds of sensors, like the received signal strength (RSS).

Another line of action will be to use a different base model for the predictive modeling. As shown in the $r^{2}$ analysis performed, discussed in Section 7, the maximum wind speed is not a linear phenomenon to model. A linear regression model with a gradient descent strategy has been used mainly as a baseline starting point, since that combination is a very well-known and easy to understand approach. The flexible modeling of the proposed methodology, using the incremental and adaptable strategy, compensates the linearity of these models. However, there are other regression models, for example support-vector-machine-based regression, which can cope with the nonlinearity of the modeled phenomenon. The authors plan to extend this research by tweaking the learning routines of these methodologies so that they become adaptable and incremental.

Finally, the authors are also considering the comparison of their results to those that can be obtained from other models and weather forecasting services, such as the one provided by the European Centre for Medium-Range Weather Forecasts (ECMWF).

## Figures and Tables

**Table 1:** AEMET Weather Stations in the Canary Islands (ID code (IDEMA), location, and island).

IDEMA	UBI	ISLAND	IDEMA	UBI	ISLAND
C018J	TIAS-LAS VEGAS	LANZAROTE	C457I	VICTORIA-DEPÓSITO MARRERO	TENERIFE
C019V	YAIZA-PLAYA BLANCA	LANZAROTE	C458A	TACORONTE-A S.E.A.	TENERIFE
C029O	LANZAROTE/AEROPUERTO	LANZAROTE	C459Z	PUERTO DE LA CRUZ	TENERIFE
C038N	HARÍA-CEMENTERIO	LANZAROTE	C469N	SILOS-DEPURADORA	TENERIFE
C048W	TINAJO-LOS DOLORES	LANZAROTE	C611E	SAN MATEO (CORRAL DE LOS JUNCOS)	GRAN CANARIA
C117A	PUNTAGORDA	LA PALMA	C612F	TEJEDA-CRUZ DE TEJEDA	GRAN CANARIA
C117Z	TIJARAFE-MIRADOR TIME	LA PALMA	C614H	TEJEDA CASCO	GRAN CANARIA
C126A	EL PASO-C.F.	LA PALMA	C619X	AGAETE-CASCO	GRAN CANARIA
C129V	FUENCALIENTE-SALINAS	LA PALMA	C619Y	LA ALDEA DE SAN NICOLAS	GRAN CANARIA
C129Z	TAZACORTE	LA PALMA	C623I	SAN BARTOLOME TIRAJANA (CUEVAS DEL PINAR)	GRAN CANARIA
C139E	LA PALMA/AEROPUERTO	LA PALMA	C625O	SAN BARTOLOME TIRAJANA-LOMO PEDRO ALFONSO	GRAN CANARIA
C148F	SAUCES-S.ANDRÉS-BALSA ADEYAHAME	LA PALMA	C628B	SAN NICOLAS T.-TASARTE/COPARLITA	GRAN CANARIA
C229J	PÁJARA-PUERTO MORRO JABLE	FUERTEVENTURA	C629Q	MOGAN (PUERTO RICO)	GRAN CANARIA
C239N	TUINEJE-PUERTO GRAN TARAJAL	FUERTEVENTURA	C629X	PUERTO DE MOGÁN	GRAN CANARIA
C248E	ANTIGUA-EL CARBÓN	FUERTEVENTURA	C635B	SAN BARTOLOME TIRAJANA-H.LAS TIRAJANAS	GRAN CANARIA
C249I	FUERTEVENTURA/AEROPUERTO	FUERTEVENTURA	C639M	SAN BARTOLOME TIRAJANA-C.INSULAR TURISMO	GRAN CANARIA
C258K	LA OLIVA (CARRETERA DEL COTILLO)	FUERTEVENTURA	C639U	SAN BARTOLOME TIRAJANA (EL MATORRAL)	GRAN CANARIA
C259X	LA OLIVA-PUERTO DE CORRALEJO	FUERTEVENTURA	C648C	AGÜIMES-EL MILANO	GRAN CANARIA
C314Z	VALLEHERMOSO-ALTO IGUALERO	LA GOMERA	C648N	TELDE-CENTRO FORESTAL DORAMAS	GRAN CANARIA
C317B	AGULO-JUEGO BOLAS	LA GOMERA	C649I	LAS PALMAS DE GRAN CANARIA/GANDO	GRAN CANARIA
C319W	VALLEHERMOSO-DAMA	LA GOMERA	C649R	TELDE-MELENARA	GRAN CANARIA
C328W	HERMIGUA-DEPÓSITO AYUNTAMIENTO	LA GOMERA	C656V	TEROR-OSORIO	GRAN CANARIA
C329Z	SAN SEBASTIÁN DE LA GOMERA	LA GOMERA	C658X	LAS PALMAS G.C.-TAFIRA/ZURBARÁN	GRAN CANARIA
C406G	CAÑADAS PARADOR	TENERIFE	C659H	LAS PALMAS G.C. SAN CRISTÓBAL	GRAN CANARIA
C419X	ADEJE-CALDERA B	TENERIFE	C659M	LAS PALMAS DE GC. PLAZA DE LA FERIA	GRAN CANARIA
C428T	ARICO-DEPURADORA LA DEGOLLADA	TENERIFE	C665T	VALLESECO	GRAN CANARIA
C429I	TENERIFE/SUR	TENERIFE	C668V	AGAETE - SUERTE ALTA	GRAN CANARIA
C430E	IZAÑA	TENERIFE	C669B	ARUCAS-BAÑADEROS	GRAN CANARIA
C438N	CANDELARIA-DEPOSITO CUEVECITAS	TENERIFE	C689E	MASPALOMAS	GRAN CANARIA
C439J	TENERIFE-GÜIMAR	TENERIFE	C839X	TEGUISE LA GRACIOSA-HELIPUERTO	LA GRACIOSA
C446G	LAS MERCEDES-LLANO LOS LOROS	TENERIFE	C916Q	PINAR-DEPÓSITO	EL HIERRO
C447A	TENERIFE/LOS RODEOS	TENERIFE	C925F	SAN ANDRÉS-DEPÓSITO CABILDO	EL HIERRO
C449C	SANTA CRUZ DE TENERIFE	TENERIFE	C929I	EL HIERRO/AEROPUERTO	EL HIERRO
C449F	ANAGA-COL. REP. ARGENTINA	TENERIFE	C939T	SABINOSA-BALNEARIO	EL HIERRO

## Abbreviations

The following abbreviations are used in this manuscript:

EU	European Union
AEMET	Agencia Estatal de Meteorología
r.p.m.	Revolutions per minute
IoE	Internet of Everything
VMAX10m	Maximum wind speed measured with a 10-min sampling period
MSE	Mean squared error
ISTAC	Canary Island Statistics Institute
